# Systemic immune-inflammation index as a novel biomarker for predicting surgical site infection in people living with HIV: a multicenter, retrospective cohort study

**DOI:** 10.3389/fcimb.2026.1529202

**Published:** 2026-02-06

**Authors:** Shuo Gong, Bo Liu, Xin Li, Wei Guo, Liqiang Hu, Qiang Zhang, Changsheng Yang, Yanguo Wang

**Affiliations:** 1 Department of Orthopedic and Soft Tissue Surgery, Shandong Cancer Hospital and Institute, Shandong First Medical University, Shandong Academy of Medical Sciences, Jinan, Shandong, China; 2 Department of Spine Surgery, Qilu Hospital of Shandong University (Qingdao), Cheeloo College of Medicine, Shandong University, Qingdao, China; 3 Department of Orthopaedics, Beijing Ditan Hospital, Capital Medical University, National Center for Infectious Diseases, Beijing, China; 4 Department of Surgery, Chengdu Public Health Clinical Medical Center, Chengdu, Sichuan, China; 5 Department of Orthopaedics, Changsha First Hospital, Xiangya Medical College, Changsha, Hunan, China

**Keywords:** a multicenter cohort study, biomarker, HIV, risk factor, surgical site infections, systemic immune-inflammation index

## Abstract

**Purpose:**

The Systemic Immune-Inflammation Index (SII) shows promise as a biomarker to assess immune status and inflammation, but its utility in predicting surgical site infections (SSIs) among HIV-infected patients remains underexplored. To evaluate SII’s predictive value for SSI risk in HIV-positive surgical patients in China, suggesting an effective clinical tool for this population.

**Methods:**

This multicenter retrospective cohort study included HIV-infected patients with fractures from three hospitals. Baseline data on demographics, HIV metrics, comorbidities, and surgical details were collected. Univariate and multivariate logistic regression analyses examined the relationship between preoperative SII and postoperative SSIs, adjusting for potential confounders like age, gender, CD4 count, viral load, and comorbidities.

**Results:**

Of 338 HIV patients, 36 (10.65%) developed postoperative SSIs. SSI patients had significantly higher SII levels. Bivariate logistic regression analysis showed that HIV viral load, open fracture, albumin, CD4, CD4/CD8 ratio and SII were risk factors for surgical site infection in HIV-positive patients. Multivariate analysis confirmed SII as an independent predictor of SSI (OR = 3.28, 95% CI = 2.07–5.54). SII showed good discriminatory performance (AUC = 0.810) and performed better than the CD4/CD8 ratio (AUC = 0.689), which was included as a representative immune-status marker. Subgroup analyses validated SII’s stability across patient subsets. Further, smooth curve fitting and RCS analysis showed that there was still a linear correlation between SII and surgical site infection in different subgroups of CD4 and HIV viral load (P for nonlinear > 0.05).

**Conclusions:**

The SII may serve as a clinically accessible and cost-effective biomarker for identifying HIV-infected patients at increased risk of SSI. Incorporating preoperative SII assessment could support perioperative risk stratification and management. This novel approach has implications for optimizing patient care for HIV-positive surgical populations.

## 1 Introduction

Surgical site infection (SSIs) is defined as a surgery-related infection that occurs within 30 days of surgery without implant placement or within 1 year of implant placement (Keely Boyle et al., 2018). They are among the most common healthcare-associated infections, contributing significantly to patient morbidity, extended hospital stays, and increased healthcare costs. SSIs can range from superficial infections involving the skin to more severe infections that affect tissues under the skin, organs, or implanted material. The risk of developing an SSI is influenced by several factors, including the surgical procedure, the patient’s underlying health condition, and the duration of the operation (Korol et al., 2013). Despite advances in surgical techniques and infection control practices, SSIs remain a substantial concern. They not only lead to prolonged hospitalization and additional surgical interventions but also increase the overall burden on healthcare systems due to the need for long-term antibiotic therapy and the increased cost of care (Tevis et al., 2016). The prevention and management of SSIs are pivotal in surgical care, requiring an approach that includes stringent sterility protocols, appropriate antibiotic use, and careful monitoring of patients’ postoperative recovery (Allegranzi et al., 2016; Mellin-Olsen et al., 2017). Recognizing the factors that contribute to the risk of SSIs and identifying patients at higher risk are crucial steps in minimizing the incidence of these infections and improving patient outcomes.

For HIV-infected patients, the risk of SSIs is particularly concerning. The compromised immune system in these individuals makes them more susceptible to infections, including SSIs. The advent of antiretroviral therapy (ART) has significantly improved the life expectancy and quality of life for individuals living with HIV, yet the risk of surgery complications remains high (Bahebeck et al., 2009). The literature by Kigera et al. (2012) highlights a direct correlation between HIV positivity and increased rates of SSI infection. While some biomarkers have shown promise in general surgical populations, their predictive value in HIV-infected patients remains less clear (Borges et al., 2013). Despite the availability of these predictive models, their applicability to all patient groups, especially those with compromised immune systems like HIV-infected patients, has been challenging. Traditional scoring systems often rely on general factors that may not fully capture the nuanced risks associated with specific conditions affecting the immune response. For instance, factors such as CD4 count, CD4/CD8 ratio and HIV viral load, which are pivotal in assessing the health status of HIV-infected patients, are typically not included in conventional SSI risk models (Xia et al., 2012; Liu et al., 2023a). This omission can lead to underestimation of the SSI risk in this unique patient population. The limitations of existing predictive models for SSIs underscore the need for innovative approaches that can provide more accurate risk stratification for HIV-infected patients. As such, research aimed at identifying reliable predictors of SSIs, especially in vulnerable populations such as HIV-infected patients, is of paramount importance.

The Systemic Immune-Inflammation Index (SII) was calculated from peripheral blood counts of neutrophils (Neu), lymphocytes (Lym), and platelets (PLT) (Luo et al., 2019). Neutrophils reflect the body’s immediate response to infection, lymphocytes represent the adaptive immune response crucial for long-term immunity, and platelets play roles in both clot formation and inflammation (Rosales, 2018; Rossaint et al., 2018; Nicolai and Massberg, 2020). The calculation of SII provides a numeric value that mirrors the patient’s systemic immune and inflammatory status. SII has been demonstrated to correlate with outcomes in various cancers, including hepatocellular carcinoma and colorectal cancer (Hu et al., 2014; Xie et al., 2018). Studies have shown that elevated SII values are associated with poorer survival rates, highlighting its potential as a marker for systemic inflammation and immune suppression (Zhong et al., 2017). Furthermore, it is able to reflect a wider range of systemic immune and inflammatory states, suggesting that its uses may extend well beyond oncology (Wang et al., 2021; Li et al., 2022; Qin et al., 2022; Liu et al., 2023b). The introduction of SII as a potential predictive tool for surgical site infections (SSIs), especially in patients with compromised immune systems like those infected with HIV, marks a significant advancement. Its basis on easily accessible blood parameters makes SII an attractive option for clinicians seeking to assess SSI risk with greater precision. This is particularly relevant for HIV-infected patients, whose altered immune responses necessitate a approach to risk evaluation.

HIV causes a decrease in the body’s CD4 cell count and immunosuppression, which leads to an increased risk of perioperative complications in this population. Therefore, there is an urgent need for more effective SSI prediction tools for HIV-infected patients. The primary aim of this multicenter study is to validate the Systemic Immune-Inflammation Index (SII) as a predictive biomarker for surgical site infections (SSIs) in PLWH. By involving multiple centers, the study is designed to capture a broad patient demographic, thereby enhancing the universality and applicability of the results. And it will critically assess the predictive accuracy of SII in relation to traditional SSI risk factors, aiming to establish SII’s potential as a more accurate predictor for PLWH. our study is predicated on the hypothesis that the Systemic Immune-Inflammation Index (SII) serves as a crucial biomarker for predicting the risk of surgical site infections (SSIs) in HIV-infected patients, with the expectation that elevated SII values are indicative of an increased risk of SSIs.

## 2 Materials and methods

### 2.1 Study design and participants

We conducted a multicenter retrospective cohort study, collecting and analyzing data from HIV-positive patients with fractures treated at three Chinese healthcare centers: Beijing Ditan Hospital, Changsha First Hospital, and Chengdu Public Health Medical Center. The study protocol was reviewed and approved by the Ethics Committee of Beijing Ditan Hospital, Capital Medical University. All procedures involving human participants were carried out in compliance with the ethical standards of the institutional and/or national research committee and with the 1964 Helsinki Declaration and its later amendments or comparable ethical standards.

The inclusion criteria for this study were as follows: (1) Patients diagnosed with HIV infection and fractures; (2) Patients who underwent surgical treatment for their fractures; (3) Patients who received follow-up care within 3 to 12 months after surgery; and (4) Patients aged between 19 and 85 years.

**Figure 1:**
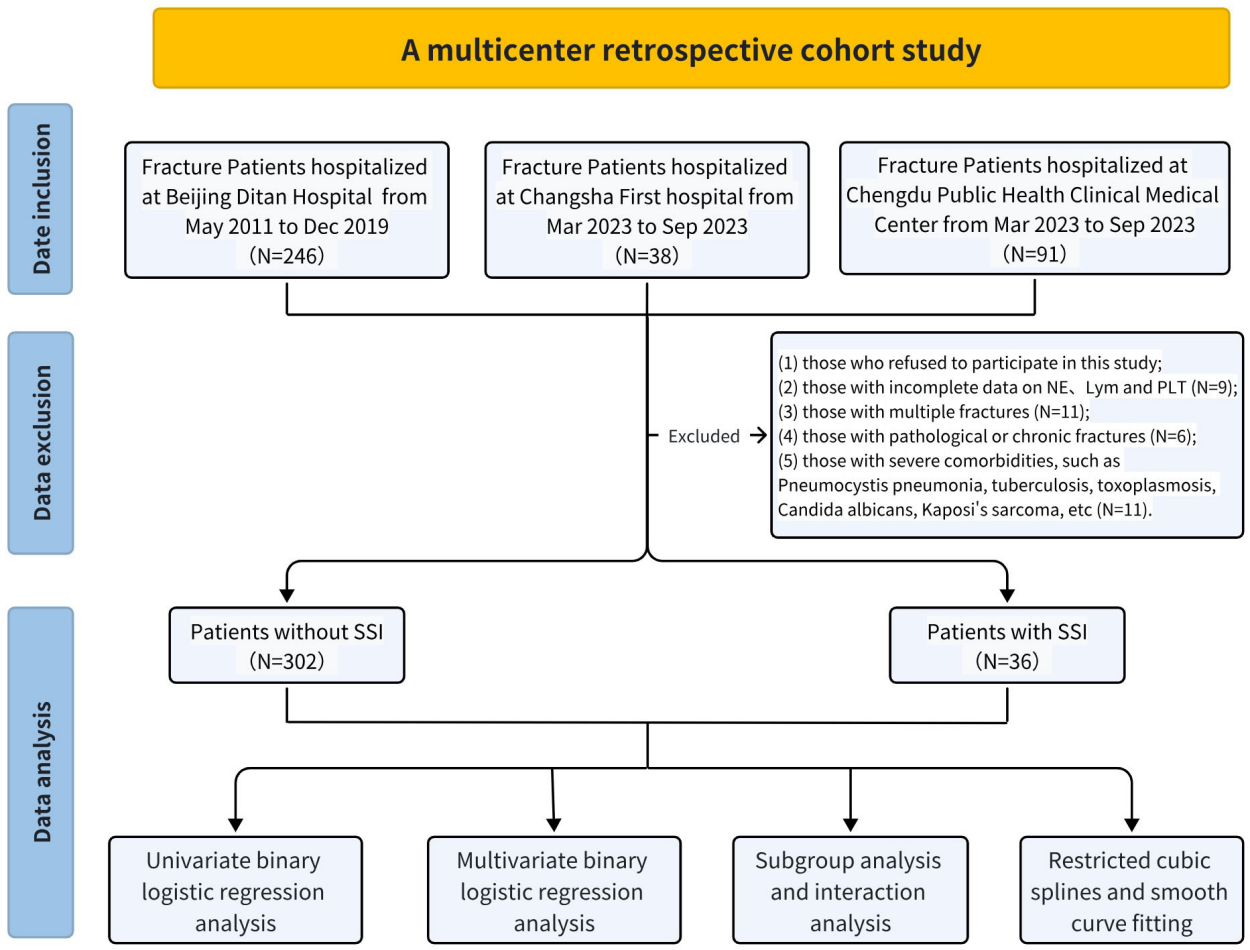
Flow chart of this study.

The exclusion criteria were: (1) Patients who declined to participate in the study (N = 3); (2) Patients with incomplete data on neutrophil, lymphocyte, and platelet counts (N = 6); (3) Patients presenting with multiple fractures (N = 11); (4) Patients with pathological or old fractures (N = 6); and (5) Patients with serious complications, such as Pneumocystis pneumonia, tuberculosis, toxoplasmosis, Candida albicans infection, Kaposi’s sarcoma, or other severe comorbidities (N = 11). The initial cohort consisted of 375 HIV-positive patients with fractures, of whom 37 were excluded based on the aforementioned criteria (**Figure 1**). The final study cohort comprised 338 patients.

### 2.2 Blood sample collection and processing

Fasting blood samples were obtained from study participants by venipuncture, using EDTA-containing Vacutainer tubes were used for routine complete blood count testing and flow cytometry for T-cell subset measurement. Serum samples were allowed to clot at room temperature for 45 minutes before being centrifuged at 3000 g for 10 minutes. Following centrifugation, serum aliquots were stored at -80°C until further analysis.

### 2.3 Measurement of HIV viral load and T lymphocyte subsets

HIV viral load in plasma was quantified using the Abbott RealTime HIV viral load assay (m2000sp, Abbott Molecular, IL, USA), with a lower detection limit of 40 copies/mL. Absolute CD4 cell counts in whole blood were determined by standard flow cytometry using a Beckman Coulter Navios instrument (Beckman, San Jose, CA, USA). Flow cytometry was used to measure CD4+ and CD8+ T-cell counts as part of routine HIV monitoring. T cell subsets were identified using fluorochrome-conjugated monoclonal antibodies procured from BD Biosciences, San Jose, CA: anti-CD4 FITC (clone RPA-T4, RRID: AB_2562052) for T helper cells and anti-CD8 AF700 (clone RPA-T8, RRID: AB_396953) for cytotoxic T cells. Cells were incubated with the antibodies for 15 minutes at room temperature in the dark, washed, and then analyzed using a Beckman Coulter Navios flow cytometer (Beckman, San Jose, CA, USA). The percentages of T helper and cytotoxic T cells were determined based on their positive surface expression of CD4 and CD8, respectively, and were reported as a proportion of the total gated lymphocyte population.

### 2.4 Assessment of systemic immune-inflammation index

Preoperative complete blood count (CBC) parameters were obtained from peripheral venous blood samples collected within 24 hours before surgery as part of routine clinical care. Neutrophil (Neu), lymphocyte (Lym), and platelet (PLT) counts were extracted from the hospital information system and used to calculate the Systemic Immune-Inflammation Index (SII) according to the standard formula:


$$SII\;\left(*10^{9}/L\right)=\frac{N\text{e}u\;\left(*10^{12}/L\right)\times Plt\;\left(*10^{9}/L\right)}{Lym\;\left(*10^{12}/L\right)}$$

Importantly, SII was calculated exclusively based on routine CBC parameters and was not derived from cell sorting or flow cytometry (FACS) assays.

**Figure 2:**
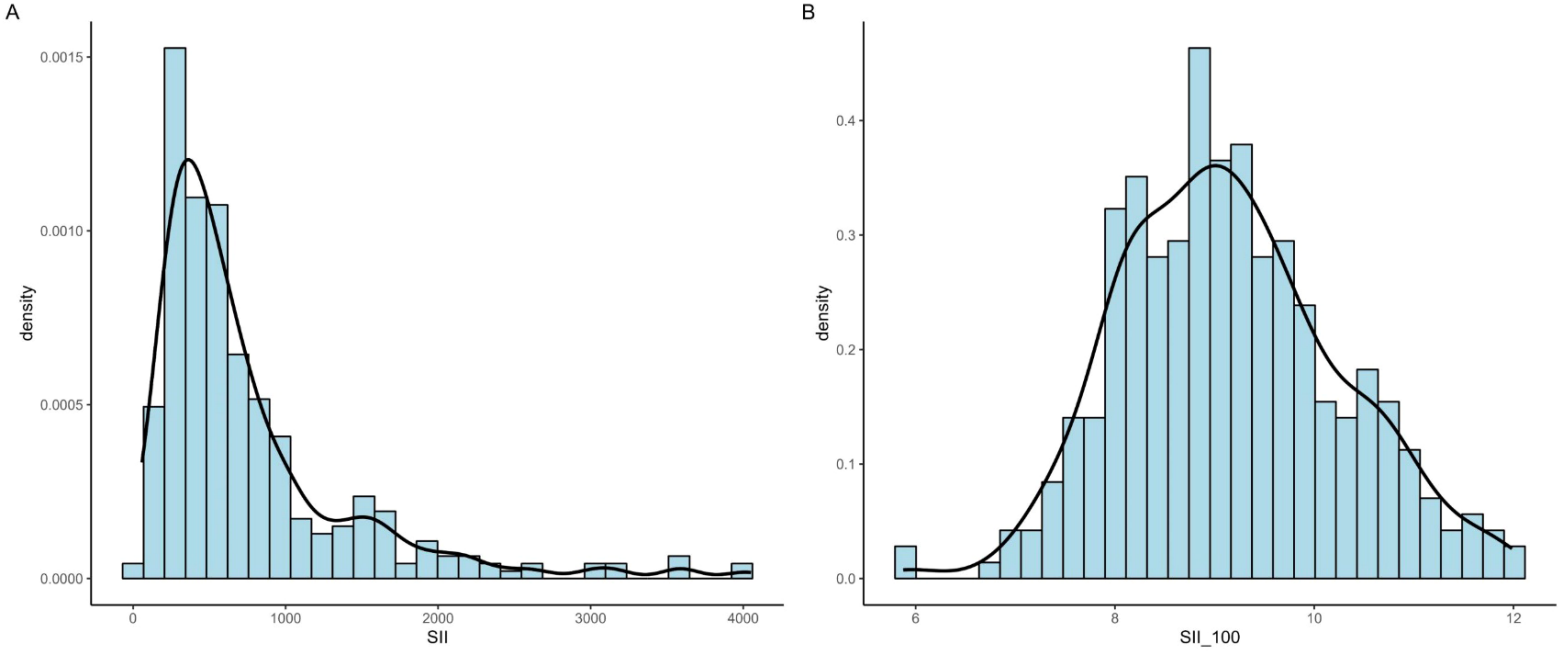
Distribution of SII before and after log2 transformation. **(A)** The distribution of preoperative SII values was right-skewed. **(B)** After log2 transformation, the distribution approximated normality.

This calculation utilized counts of neutrophils (Neu), lymphocytes (Lym), and platelets (PLT) obtained from peripheral blood samples, reflecting the patients’ immune and inflammatory status. However, we found that the SII distribution was right-skewed (**Figure 2A**); after log2 transformation, the distribution converted to normal (**Figure 2B**).

### 2.5 Assessment of surgical site infections

In this study, the primary outcome variable was the occurrence of surgical site infections (SSIs). SSIs were defined and classified according to the criteria established by the American Academy of Orthopaedic Surgeons (AAOS) (Berríos-Torres et al., 2017; McLaren and Lundy, 2019). SSI was considered to be any infection related to the surgical procedure that occurred within 30 days after surgery in the absence of an implant, or within 1 year after surgery when an implant was present. Implants included, but were not limited to, fracture internal fixation systems, vertebral pedicle screws, intervertebral fusion devices, artificial intervertebral discs, and prostheses. The classification of SSIs encompassed superficial incisional infections, deep incisional infections, and organ/space infections. Superficial incisional SSIs involved only the skin and subcutaneous tissue, while deep incisional SSIs affected deeper soft tissues, and organ/space SSIs extended beyond the surgical incision to involve any part of the anatomy manipulated during the procedure.

The consistent application of this well-defined criteria across all participating centers ensured the reliable identification of SSIs and enabled an accurate evaluation of the predictive value of the Systemic Immune-Inflammation Index (SII). The diagnosis of SSIs was made by the treating surgeons based on clinical signs and symptoms, such as localized pain, tenderness, swelling, redness, warmth, or purulent discharge from the surgical site. Microbiological culture results, when available, were used to support the diagnosis and guide antimicrobial therapy.

### 2.6 Data collection and covariates

To comprehensively assess the potential risk factors for surgical site infections (SSIs) in HIV-positive patients, we collected a wide range of clinical data, including patient demographics, HIV-related health indicators, comorbidities, and surgical characteristics. Demographic information included age (categorized as<30 and ≥30 years), sex (male and female), duration of HIV infection (<2 and ≥2 years), antiretroviral therapy (ART) status (yes or no), and presence of comorbidities such as hepatitis and diabetes mellitus. Preoperative laboratory parameters were also recorded, including hemoglobin levels (Hb, <120 and ≥120 g/L), serum albumin concentrations (<40 and ≥40 g/L), CD4+ T cell counts (<200 and ≥200 cells/µl), CD8+ T cell counts (<400 and ≥400 cells/µl), HIV viral loads (categorized as undetectable, <20, 000, and ≥20, 000 copies/ml), as well as neutrophil, lymphocyte, and platelet counts. Surgical factors were also considered, such as fracture site (upper limbs, lower limbs, or spine), fracture type (closed or open), anesthesia type (general or local), and estimated blood loss during the procedure (<400 and ≥400 ml). T lymphocyte subpopulations were quantified using flow cytometry, a technique that allows for the direct measurement of absolute CD4+ T cell counts and the determination of the relative proportion of CD4+ T cells within the total lymphocyte population. This method provides a reliable assessment of the patient’s immune status and has been widely used in HIV monitoring and research.

All clinical data were extracted from the hospital information system (HIS) medical records by trained research staff. To ensure data accuracy and reliability, each data point was independently reviewed and verified by at least two resident physicians involved in the study. In cases of discrepancies, the medical records were re-examined, and consensus was reached through discussion among the research team. By gathering such a comprehensive set of clinical data, we aimed to identify the most relevant risk factors for SSIs in our HIV-positive patient cohort and to develop a robust predictive model based on the Systemic Immune-Inflammation Index (SII) and other key variables.

### 2.7 Antibiotic prophylaxis protocol

In this study, all patients received prophylactic antibiotics (cefazolin or cefuroxime) intravenously during surgery and for 48 hours postoperatively, according to institutional guidelines. These first-generation cephalosporins were chosen for their broad-spectrum activity against common pathogens associated with surgical site infections (SSIs) in orthopedic procedures, particularly Gram-positive bacteria like Staphylococcus aureus and Staphylococcus epidermidis. The dosing regimen consisted of 1.5 grams administered intraoperatively, followed by 1.5 grams every 8 hours for 48 hours postoperatively. This schedule was designed to maintain therapeutic antibiotic concentrations at the surgical site throughout the perioperative period. The 48-hour duration was based on evidence showing no additional benefits in reducing SSI rates with prolonged prophylaxis, while minimizing the risk of adverse effects and antibiotic resistance. Adherence to the prophylaxis protocol was closely monitored by surgical and nursing staff, and any deviations were reviewed by the infection control team to ensure optimal patient care.

### 2.8 Statistical analysis

Data analysis was performed using R version 4.1.3 software (https://www.R-project.org), with a P-value less than 0.05 considered statistically significant. Continuous variables were presented as mean ± standard deviation (SD) or interquartile range (IQR), and differences between groups were assessed using one-way ANOVA or Kruskal-Wallis U test, as appropriate. Categorical variables were expressed as percentages (n, %), and differences between groups were compared using the Chi-squared test or Fisher’s exact test. The difference in SII between patients with and without SSI was assessed using an independent t-test for normally distributed data. If data were not normally distributed, a Mann-Whitney U test was performed. The variance in SII between the groups was substantial, indicating heterogeneity in inflammatory responses.

To identify potential risk factors for SSIs in the study cohort, univariate binary logistic regression models were employed, incorporating SII and other clinical variables such as HIV viral load, CD4 count, and the presence of comorbidities. This comparison was designed to evaluate whether SII provides additional discriminatory information compared with a conventional immune-status marker in HIV care, rather than to benchmark all established clinical or surgical risk factors.

To further investigate the relationship between SII and SSIs while controlling for potential confounders, we constructed a series of multivariable logistic regression models with increasing levels of adjustment. The crude model did not adjust for any covariates, while model 1 adjusted for age and gender. Model 2 additionally adjusted for hepatitis (HBV and/or HCV), diabetes, HIV viral load, duration of HIV infection, and ART status, while model 3 further included surgical site, fracture type, anesthesia type, and estimated blood loss as covariates. Finally, model 4 adjusted for all the covariates in model 3 plus serum albumin, hemoglobin, CD4, and CD8 counts.

Subgroup analyses were performed to assess the stability of the findings across different patient subsets and to explore potential interactions among various predictors. These analyses provided insights into the generalizability of the results and helped identify any effect modification by key clinical characteristics. To examine the potential nonlinear relationship between SII and SSIs, we employed restricted cubic splines (RCS) and smooth curve fitting techniques. These methods allowed for a flexible modeling of the association, capturing any potential nonlinearities or threshold effects. The smooth curve fitting was performed using generalized additive models (GAMs) with a penalized spline function to estimate the nonlinear relationship. All statistical analyses were conducted using appropriate R packages, such as ‘glm’ for logistic regression, ‘pROC’ for ROC analysis, and ‘rms’ and ‘mgcv’ for RCS and GAM fitting, respectively.

No additional cell sorting or FACS experiments were performed specifically for SII, as SII is a composite index derived from routine CBC parameters.

## 3 Results

### 3.1 Demographic characteristics of HIV-positive patients with or without surgical site infection

In our study, 338 HIV-infected patients diagnosed with fractures were included. In total, 338 HIV-infected patients with fractures were included: 226 (63.9%) from Beijing Ditan Hospital, 86 (25.4%) from Chengdu Public Health Clinical Medical Center, and 36 (10.6%) from Changsha First Hospital (**Table 1**). Among the 36 patients who developed SSI, 23 (63.89%) were from Beijing Ditan Hospital, 9 (25.00%) from Chengdu Public Health Clinical Medical Center, and 4 (11.11%) from Changsha First Hospital (**Table 1**). The distribution of SSI cases across hospitals was comparable to that of non-SSI cases, and no significant difference was observed across centers (P = 0.994).

**Table 1:** Baseline characteristics of HIV-positive fracture patients in Beijing Ditan Hospital, Changsha First People's Hospital, and Chengdu Public Health Clinical Medical Center.

Variables	Overall (N=338)	Beijing (N=216)	Changsha (N=36)	Chengdu (N=86)	P-value
SSI (%)					
No	302 (89.35)	193 (89.35)	32 (88.89)	77 (89.53)	0.994
Yes	36 (10.65)	23 (10.65)	4 (11.11)	9 (10.47)	
Age (%, years)					
<30	48 (14.20)	39 (18.06)	3 (8.33)	6 (6.98)	0.026
≥30	290 (85.80)	177 (81.94)	33 (91.67)	80 (93.02)	
Gender (%)					
Female	41 (12.13)	22 (10.19)	7 (19.44)	12 (13.95)	0.242
Male	297 (87.87)	194 (89.81)	29 (80.56)	74 (86.05)	
Hepatitis (%)					
No	269 (79.59)	172 (79.63)	31 (86.11)	66 (76.74)	0.504
Yes	69 (20.41)	44 (20.37)	5 (13.89)	20 (23.26)	
Diabetes (%)					
No	278 (82.25)	188 (87.04)	22 (61.11)	68 (79.07)	0.001
Yes	60 (17.75)	28 (12.96)	14 (38.89)	18 (20.93)	
ART (%)					
No	44 (13.02)	29 (13.43)	6 (16.67)	9 (10.47)	0.622
Yes	294 (86.98)	187 (86.57)	30 (83.33)	77 (89.53)	
HIV_duration (%)					
Diagnosed_at_fracture	92 (27.22)	92 (42.59)	0 (0.00)	0 (0.00)	<0.001
<2_year	37 (10.95)	34 (15.74)	1 (2.78)	2 (2.33)	
≥2_year	209 (61.83)	90 (41.67)	35 (97.22)	84 (97.67)	
RNA load (%, copes/ml)					
Not_detected	210 (62.13)	117 (54.17)	27 (75.00)	66 (76.74)	<0.001
<20000	83 (24.56)	59 (27.31)	7 (19.44)	17 (19.77)	
≥20000	45 (13.31)	40 (18.52)	2 (5.56)	3 (3.49)	
Site (%)					
Leg	238 (70.41)	157 (72.69)	24 (66.67)	57 (66.28)	0.169
Arm	66 (19.53)	43 (19.91)	5 (13.89)	18 (20.93)	
Spine	34 (10.06)	16 (7.41)	7 (19.44)	11 (12.79)	
Open_fracture (%)					
No	316 (93.49)	203 (93.98)	32 (88.89)	81 (94.19)	0.495
Yes	22 (6.51)	13 (6.02)	4 (11.11)	5 (5.81)	
Type_of_anesthesia (%)					
RA	242 (71.60)	137 (63.43)	30 (83.33)	75 (87.21)	<0.001
GA	96 (28.40)	79 (36.57)	6 (16.67)	11 (12.79)	
Bleed (%, ml)					
<400	231 (68.34)	118 (54.63)	35 (97.22)	78 (90.70)	<0.001
≥400	107 (31.66)	98 (45.37)	1 (2.78)	8 (9.30)	
Albumin (%, g/L)					
<40	104 (30.77)	80 (37.04)	6 (16.67)	18 (20.93)	0.004
≥40	234 (69.23)	136 (62.96)	30 (83.33)	68 (79.07)	
Hb (%, g/L)					
<120	80 (23.67)	54 (25.00)	10 (27.78)	16 (18.60)	0.413
≥120	258 (76.33)	162 (75.00)	26 (72.22)	70 (81.40)	
CD4 (%, Cells/ul)					
<200	58 (17.16)	40 (18.52)	7 (19.44)	11 (12.79)	0.457
≥200	280 (82.84)	176 (81.48)	29 (80.56)	75 (87.21)	
CD8 (%, Cells/ul)					
<400	50 (14.79)	32 (14.81)	5 (13.89)	13 (15.12)	0.985
≥400	288 (85.21)	184 (85.19)	31 (86.11)	73 (84.88)	
Ratio CD4/CD8 [mean (SD)]	0.63 (0.45)	0.59 (0.38)	0.63 (0.42)	0.74 (0.58)	0.038
Ne (mean (SD), 10^9/L)	4.47 (2.29)	4.27 (1.97)	4.51 (2.22)	4.94 (2.94)	0.075
Lym (mean (SD), 10^9/L)	1.68 (0.76)	1.63 (0.68)	1.63 (0.63)	1.85 (0.94)	0.061
Plt (mean (SD), 10^9/L)	227.76 (69.06)	228.75 (72.97)	232.19 (60.62)	223.43 (62.46)	0.768
SII (mean (SD), 10^9/L)	761.67 (679.58)	744.65 (618.83)	791.07 (707.00)	792.10 (809.00)	0.83

SSI, Surgical Site Infection; HIV, Human Immunodeficiency Virus; ART, Active Antiretroviral Therapy; GA, general anesthesia; RA, Reginol anesthesia; Hb, hemoglobin; Ne, neutrophils; Lym, lymphocyte; Plt, platelet; SII, systemic immune-inflammation index.

**Figure 3:**
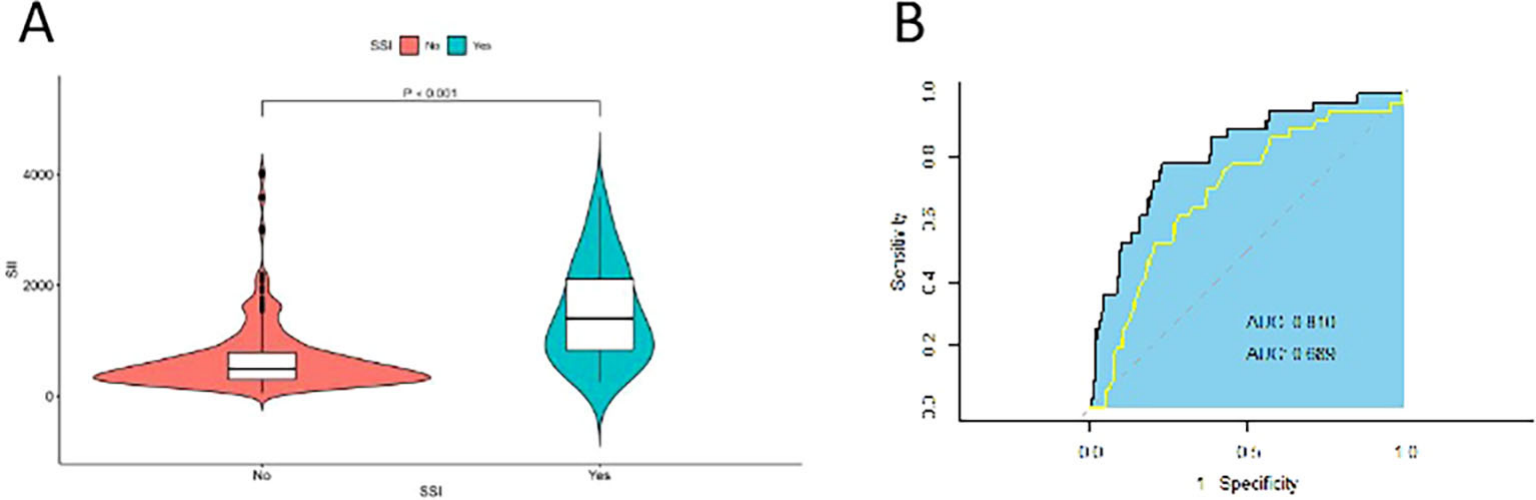
Comparison of SII and CD4/CD8 ratio in predicting SSI risk. **(A)** Violin plot of the SII difference between patients with SSI and those without. Statistical significance was assessed using the Mann-Whitney U test due to the non-normal distribution of SII values. **(B)** Receiver operating characteristic (ROC) curves and AUC values for SII and CD4/CD8 ratio in predicting SSI. The AUCs were compared using DeLong’s test to assess the performance of each predictor.

The study flow diagram, including patient screening, exclusion criteria, and the final analytic cohort, is presented in **Figure 1**. 36 (10.65%) developed surgical site infections (SSIs) during the perioperative period. The SII of the group without surgical site infection was 607.5 ± 460.2, while the SII of the group with surgical site infection was 1317.8 ± 837.1; As shown in **Figure 3A**, SII levels were significantly higher in patients with surgical site infection than in those without surgical site infection. Upon reviewing the baseline demographics and clinical characteristics, no significant differences were noted in age and gender distribution between patients who developed SSIs and not. However, a notable distinction was observed in the Neu levels, Lym levels, HIV viral load, and CD4 counts between the two groups (**Table 1**).

There was a significant difference in SII between the groups with and without SSI; however, the variance within both groups was relatively large (460.2 for the non-SSI group, and 837.1 for the SSI group), suggesting considerable heterogeneity in inflammatory responses across individuals. This variability may be due to the diverse factors influencing immune function and inflammation in HIV-infected patients. As shown in **Figure 2A**, the preoperative SII values were right-skewed. Therefore, SII was log2-transformed to improve normality, and the transformed distribution approximated normality (**Figure 2B**).

### 3.2 Binary univariate logistics analysis and SII’s predictive ability

The univariate logistic regression analysis identified several factors associated with an increased risk of SSIs (**Table 2**). These included a higher HIV viral load (odds ratio [OR] = 5.51, 95% CI = 2.3-13.2), the presence of open fractures (OR = 4.62, 95% CI = 1.65-11.9), lower albumin levels (OR = 0.4, 95% CI = 0.2-0.8), reduced CD4 counts (OR = 0.18, 95% CI = 0.08-0.37), lower CD4/CD8 ratios (OR = 0.26, 95% CI = 0.08-0.74), and higher SII values (OR = 3.09, 95% CI = 2.15-4.6). These initial findings underscored the potential relevance of various clinical parameters, including SII, in identifying patients at heightened risk for SSIs.

**Table 2:** The univariate logistic regression analysis of surgical site infection risk in HIV patients.

Characteristic	N	OR^1^	95% CI^1^	P-value
Age (years)				0.955
<30	48	—	—	
≥30	290	1.03	0.41, 3.14	
Gender				0.397
Female	41	—	—	
Male	297	0.66	0.27, 1.84	
Hepatitis				0.054
No	269	—	—	
Yes	69	2.15	0.99, 4.48	
Diabetes				0.247
No	278	—	—	
Yes	60	1.64	0.69, 3.58	
ART				0.871
No	44	—	—	
Yes	294	0.92	0.36, 2.82	
HIV_duration				0.412
Diagnosed_at_fracture	92	—	—	
<2_year	37	1.43	0.46, 4.09	
≥2_year	209	0.74	0.34, 1.67	
HIV RNA load (copes/ml)				**<0.001**
Not_detected	210	—	—	
<20000	83	2.32	0.98, 5.41	
≥20000	45	5.51	2.30, 13.2	
Site				0.330
Leg	238	—	—	
Arm	66	0.70	0.23, 1.76	
Spine	34	1.83	0.63, 4.60	
Open_fracture				**0.005**
No	316	—	—	
Yes	22	4.62	1.65, 11.9	
Type_of_anesthesia				0.288
RA	242	—	—	
GA	96	1.49	0.70, 3.04	
Bleed (ml)				0.181
<400	231	—	—	
≥400	107	1.63	0.79, 3.29	
Albumin (g/L)				**0.011**
<40	104	—	—	
≥40	234	0.40	0.20, 0.80	
Hb (g/L)				0.031
<120	80	—	—	
≥120	258	0.44	0.21, 0.92	
CD4 (cells/ul)				**<0.001**
<200	58	—	—	
≥200	280	0.18	0.08, 0.37	
CD8 (cells/ul)				0.871
<400	50	—	—	
≥400	288	1.09	0.43, 3.31	
Ratio CD4/CD8	338	0.26	0.08, 0.74	**0.009**
Neu (10^9/L)	338	1.27	1.12, 1.44	**<0.001**
Lym (10^9/L)	338	0.05	0.02, 0.13	**<0.001**
Plt (10^9/L)	338	1.00	1.0, 1.00	0.985
SII (10^9/L)	338	3.09	2.15, 4.60	**<0.001**

^1^OR = Odds Ratio, CI = Confidence Interval, SSI, Surgical Site Infection; HIV, Human Immunodeficiency Virus; ART, Active Antiretroviral Therapy; GA, general anesthesia; RA, Reginol anesthesia; Hb, hemoglobin; Ne, neutrophils; Lym, lymphocyte; Plt, platelet; SII, systemic immune-inflammation index.

Bold text indicates that a p-value < 0.05 indicates a statistically significant difference.

Furthermore, we evaluated the discriminatory performance of SII in predicting SSIs and compared it with the CD4/CD8 ratio, a commonly used immune-status marker in HIV-related clinical practice. The ROC analysis yielded an AUC of 0.810 for SII, compared with 0.689 for the CD4/CD8 ratio (**Figure 3B**). The difference between the two AUCs was statistically significant (Z = 2.1573, p=0.031), suggesting that SII may offer improved discrimination over the CD4/CD8 ratio in this cohort.

### 3.3 Binary multivariate logistics analysis of SSIs in HIV-positive fracture patients

After performing multivariate binary logistic regression analysis (As shown in **Table 3**), our results indicate that a higher SII score is associated with an increased risk of developing surgery site infection. This association was significant in our crude model (OR = 3.086; 95%CI=2.149-4.602, p<0.001), model 1 (OR = 3.078; 95%CI=2.142-4.591, p<0.001), model 2 (OR = 3.247; 95%CI=2.191-5.065, p<0.001) and model 3 (OR = 3.381; 95%CI=2.235-5.409, p<0.001). In the fully adjusted model, the positive association between SII and SSI remained stable (OR = 3.276; 95%CI=2.067-5.549, p<0.001), indicating that for every unit increase in log-SII levels, the risk of developing SSI increased by 3.276-fold.

**Table 3:** Multivariate logistic regression analysis between systemic immune inflammation index and surgical site infection.

Models	Continuous variables		Categorical variables lower SII (<533.4) *V.S.* Higher SII ( ≥ 533.4)		
	OR (95%CI)	P-value	OR (95%CI)	P-value	P for trend
Crude model	3.086(2.149, 4.602)	<0.001	9.635(3.710, 32.945)	<0.001	<0.001
Model 1	3.078(2.142, 4.591)	<0.001	9.597(3.694, 32.829)	<0.001	<0.001
Model 2	3.247(2.191, 5.065)	<0.001	12.251(4.445, 43.915)	<0.001	<0.001
Model 3	3.381(2.235, 5.409)	<0.001	13.611(4.740, 51.091)	<0.001	<0.001
Model 4	3.276(2.067, 5.549)	<0.001	10.801(3.594, 41.974)	0.004	0.004

Crudel model: No covariates were adjusted; Model 1: Age, Gender; Model 2: model 1 + Hepatitis, Diabetes, ART, HIV_duration, RNA;Model 3: model 2 + Bleed, Open_fracture, Type_of_anesthesia, Site; Model 4: model 3 + Albumin, Hb, CD4, CD8

We further transformed the SII from a continuous variable into a categorical variable (lower SII vs higher SII) for sensitivity analysis (**Table 3**). Compared with the lower SII, the risk of developing SSI in the higher SSI increased by 9.635-fold (OR = 9.635; 95%CI=3.71-32.945, p<0.001) in the crude model and 10.8-fold (OR = 10.801; 95%CI=3.594-41.974, p<0.001). That is, patients with higher SII levels exhibited a markedly increased risk of SSIs compared to those with lower SII levels, reinforcing the value of SII in preoperative risk stratification.

### 3.4 Subgroup analysis and RCS

Subgroup analysis provided additional insights, demonstrating the stability of the relationship between SII levels and SSI risk across different age (<30 and ≥30 years), gender (male and female), hepatitis (Yes and No), and diabetes mellitus (Yes and No), type of anesthesia (general or local), Hb (<120 and ≥120 g/L), albumin (<40 and ≥40 g/L), and CD4+ counts (<200 and ≥200 cells/ul) subgroups (**Table 4**).

**Table 4:** Subgroup analysis and interaction analysis between systemic immune inflammation index and risk of surgical site infection.

Variables	95% CI	P-value	P for interaction
Gender			0.594
Female	2.457(1.020,5.918)	0.045	
Male	3.221(2.116,4.902)	<0.001	
Age (years)			0.071
<30	28.928(1.462,572.339)	0.027	
≥30	2.757(1.878,4.047)	<0.001	
Hepatitis			0.807
No	3.195(2.016,5.063)	<0.001	
Yes	3.567(1.657,7.677)	0.001	
Diabetes			0.161
No	3.849(2.368,6.258)	<0.001	
Yes	2.121(1.101,4.087)	0.025	
Type_of_anesthesia			0.86
RA	3.062(1.959,4.787)	<0.001	
GA	3.312(1.557,7.042)	0.002	
Albumin (g/L)			0.787
<40	2.78(1.611,4.794)	<0.001	
≥40	3.087(1.822,5.230)	<0.001	
Hb (g/L)			0.19
<120	2.188(1.245,3.847)	0.007	
≥20	3.673(2.192,6.154)	<0.001	
CD4 (cells/ul)			0.419
<200	2.355(1.306,4.246)	0.004	
≥200	3.268(1.940,5.504)	<0.001	

**Figure 4:**
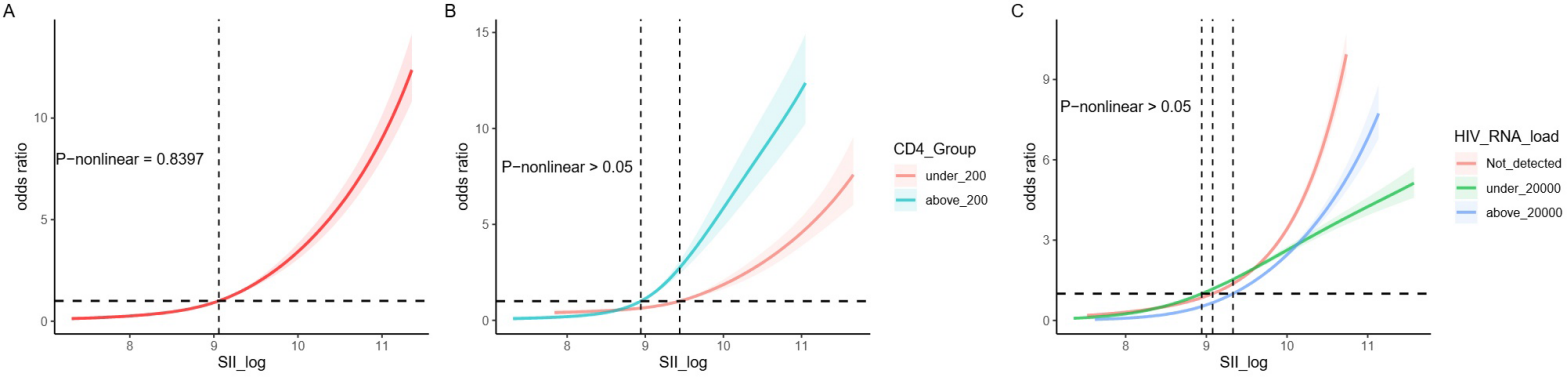
**(A)** Linear smooth fit curve between SI and SSI; **(B)** Relationship between SI and SSI across CD4 subgroups; **(C)** Relationship between SI and SSI across HIV viral load subgroups.

Moreover, as shown in **Figure 4**, a linear correlation between SII and SSIs was observed (**Figure 4A**), regardless of variations in CD4 count (<200 and ≥200 cells/ul, **Figure 4B**) and HIV viral load (undetectable, <20, 000, and ≥20, 000 copes/mL, **Figure 4C**), indicating a consistent association between systemic immune inflammation and SSI risk among HIV-positive patients with fractures. This consistency across various patient demographics and clinical profiles underscores the reliability and applicability of SII as a predictive marker for SSIs.

## 4 Discussion

Our findings suggest that SII is a clinically relevant biomarker associated with SSI risk in HIV-infected patients. As a low-cost index derived from routinely available blood parameters, SII may complement existing HIV-related immune markers and support perioperative risk stratification.The subgroup analysis and RCS conducted in our study sheds light on the nuanced relationship between the Systemic Immune-Inflammation Index (SII) and surgical site infection (SSI) risk among different patient groups, highlighting its potential for personalized medicine. This analysis reveals that SII’s predictive accuracy for SSIs remains robust across various age groups, suggesting its universal applicability among HIV-infected patients undergoing surgery. The significance of this finding lies in its affirmation of SII as a versatile biomarker that can guide clinical decision-making across a diverse patient population. By identifying specific subgroups that may have a distinct risk profile, healthcare providers can further tailor their preventive strategies and postoperative care, ensuring that interventions are as effective and targeted as possible.

Comparing our results with existing research underscores the novel application and efficacy of the Systemic Immune-Inflammation Index (SII) in predicting surgical site infection (SSI) risk among HIV-infected patients. Previous studies have primarily focused on the predictive value of SII in various cancers, where it has been shown to reflect the balance between host inflammatory and immune status effectively (Yang et al., 2018; Zhang et al., 2020; He et al., 2022). For non-cancer diseases, the latest research progress shows that SII has wide application potential in cardiovascular diseases, autoimmune diseases, metabolic syndrome and other non-cancer diseases (Ye et al., 2022). For example, in the field of cardiovascular disease, Xu et al. (2021) showed that higher SII values in the elderly were closely associated with worse prognosis of cardiovascular events. In addition, SII has shown great significance in assessing disease activity, monitoring treatment efficacy, and predicting the risk of disease recurrence (Wang et al., 2021; Li et al., 2022; Qin et al., 2022). Using the NHANES database, Liu et al. (2023b) concluded that higher levels of SII promote the development of rheumatoid arthritis.

Our study extends the utility of SII to a new domain, showcasing its potential beyond oncology by demonstrating its predictive power for SSIs in a population compromised by HIV (Liu et al., 2023a). This is particularly significant given the unique challenges in managing infections in HIV-infected patients, who are inherently at a higher risk due to their compromised immune systems (Wang et al., 2021). The findings suggest that SII, by offering a more comprehensive reflection of the body’s immune and inflammatory state, may outperform traditional markers like the CD4/CD8 ratio, which have been the mainstay for assessing immune function in HIV patients. Recent studies have shown that independent predictors of surgical site infection (SSI) in HIV-positive patients undergoing orthopedic surgery include CD4 count, erythrocyte sedimentation rate (ESR), and procalcitonin (PCT) (Ma et al., 2020). In addition, Liu et al. explored the relationship between CD4/CD8 ratio and the risk of SSI in HIV-positive adults after orthopedic surgery, finding that CD4/CD8 ratio is a specific marker of immune inflammation (Liu et al., 2023a). Previous research has underscored the importance of immune and inflammatory status in predicting surgical outcomes, yet few have specifically examined SII within this context. Our study contributes novel insights, particularly regarding the superior predictive capacity of SII over conventional markers like the CD4/CD8 ratio in the context of the Chinese HIV-infected population.This not only reinforces the value of SII as a robust, economical biomarker for clinical assessments but also opens up new avenues for its application in improving surgical outcomes and patient care strategies in populations with underlying immune system challenges.

The predictive value of the Systemic Immune-Inflammation Index (SII) in identifying the risk of surgical site infections (SSIs) among HIV-infected patients might be attributed to underlying biological mechanisms. SII integrates neutrophil, lymphocyte, and platelet counts, each reflecting different facets of the body’s immune and inflammatory responses. Neutrophils play a key role in the acute inflammatory response to pathogens, whereas lymphocytes are crucial for adaptive immunity, providing long-term defense against infections (Rosales, 2020; Lok and Clatworthy, 2021). Platelets, beyond their role in clotting, also contribute to inflammation and immune regulation (Oryan et al., 2016; Tokarz-Deptuła et al., 2021). Elevated SII levels, indicative of increased neutrophil and platelet counts relative to lymphocytes, may signal a heightened inflammatory state and altered immune response, which in turn could increase the vulnerability to SSIs. Understanding this balance between immune response and inflammation is crucial for developing targeted interventions aimed at reducing SSI risk in this population.

And recent studies have reported that higher SII levels are strongly associated with poor prognosis after surgery, as it reflects the interaction of inflammation and immune cells in the tumor microenvironment, suggesting possible inflammatory and immune escape mechanisms that may affect the risk and prognosis of surgical site infections (Katayama et al., 2021; Li et al., 2021). For example, in gastric cancer patients, where the optimal cutoff for pre-operative SII was 508.3, the 5-year overall survival rate was significantly higher in the SII low group than in the SII high group, especially in elderly and stage II patients, indicating that SII has significant prognostic value in most subgroups (He et al., 2022). In another study, high levels of SII were strongly associated with poor outcomes and short-term outcomes after surgery in patients with valvular heart disease and could be an independent predictor (Xiang et al., 2022). Thus, the ability of SII as a predictive biomarker for surgical site infection may stem from its combined effect of reflecting inflammation and immune status in the body, which are associated with poor postoperative outcomes, including increased risk of infection.

The study of the Systemic Immune-Inflammation Index (SII) as a biomarker for predicting surgical site infection (SSI) risk in HIV-infected patients holds profound significance for several reasons. ***First*,** it addresses a critical gap in current surgical care by providing a potentially more accurate tool for assessing SSI risk in a population particularly vulnerable due to compromised immune systems. The conventional methods of predicting SSIs may not fully capture the unique risks faced by HIV-infected patients, highlighting the need for more tailored approaches. *Second*, by evaluating the effectiveness of SII in this specific context, the study aims to contribute valuable insights into the interplay between systemic inflammation, immune status, and surgical outcomes. Such insights could lead to improved preoperative risk stratification, enabling healthcare providers to implement more personalized preventive measures and optimize surgical planning. *Furthermore* the adoption of SII as a predictive marker could enhance postoperative care strategies, potentially reducing the incidence of SSIs. This would not only improve patient outcomes but also decrease healthcare costs associated with the treatment of SSIs. ***Overall*,** this study represents an important step towards personalized medicine in the surgical care of HIV-infected patients. By exploring the predictive value of SII, it seeks to pave the way for more informed clinical decision-making, ultimately contributing to safer surgical procedures and better health outcomes for this vulnerable patient group.

Our study, while insightful, is not without limitations, which opens avenues for future research. One notable limitation is its retrospective design, which, despite providing valuable data, may encompass biases related to data collection and patient selection. Furthermore, the study focuses exclusively on HIV-infected patients within several specific healthcare centers in China, potentially limiting the generalizability of our findings to other populations or regions. Future studies could benefit from a prospective approach, encompassing a broader demographic to validate the predictive power of SII for SSIs across different healthcare settings and populations. Future studies should explore the applicability of SII in other surgical populations and infection types to validate its broad utility. Additionally, longitudinal studies could provide insights into how SII levels change over time with treatment and disease progression, offering a dynamic tool for ongoing patient management. Investigating the molecular mechanisms underlying the correlation between SII and infection risk could also uncover new therapeutic targets for preventing SSIs. Finally, research should aim to refine the SII calculation, potentially incorporating new biomarkers to enhance its predictive accuracy and clinical relevance.

## 5 Conclusion

In summary, we found that the Systemic Immune-Inflammation Index (SII) levels were related to the risk of SSIs. SII was independently associated with SSI risk in HIV-infected fracture patients and showed good discriminatory performance. Given its accessibility and low cost, SII may be considered as an adjunct biomarker to support preoperative risk assessment. Further prospective studies are warranted to validate its clinical utility and to compare its performance with other established predictors. We believe these findings will contribute significantly to the clinical management of HIV-infected patients, enhancing surgical outcomes and patient safety.

## Data Availability

The original contributions presented in the study are included in the article/supplementary material. Further inquiries can be directed to the corresponding author.
